# Fiber Aggregation in Nanocomposites: Aggregation Degree and Its Linear Relation with the Percolation Threshold

**DOI:** 10.3390/ma16010015

**Published:** 2022-12-20

**Authors:** Baorang Cui, Fei Pan, Bin Ding, Feng Zhang, Yong Ma, Yuli Chen

**Affiliations:** 1 Institute of Solid Mechanics, Beihang University, Beijing 100191, China; 2 School of Aeronautic Science and Engineering, Beihang University, Beijing 100191, China

**Keywords:** nanocomposites, degree of aggregation, analytical modelling, percolation threshold

## Abstract

Fiber aggregation in nanocomposites has an important effect on macroscopic electrical performance. To quantitatively evaluate its effect, an index to characterize the degree of aggregation is imperative and, ideally, it should have three features simultaneously, i.e., (1) single-parametric, dimensionless, and physically meaningful, (2) applicable to different aggregation topologies, and (3) one-to-one, corresponding to material electrical properties. However, these features remain largely unexplored. Here, we propose a new aggregation degree that is defined as the average increment of the fiber number connecting with each one when fibers aggregate from a uniform distribution state. This index is applicable to different aggregation topologies, from lump-like to network-like aggregating clusters. By geometric probability analysis and numerical validations, we demonstrate the index can be concisely expressed by the characteristic parameters of the aggregating cluster since it only depends on the local features. Interestingly, a one-to-one linear relation between the aggregation degree and the percolation threshold is found, which is independent of the distribution law of the fibers. This work may provide a guide to the property characterization, performance prediction, and material design of nanocomposites, and give physical insight into the understanding of systems with similar non-uniform distributions.

## 1. Introduction

Nanofiber-reinforced composites have extensive applications in many fields, e.g., antennas and solar sails for spacecraft [1], flexible wearable electronics [2], high-efficiency solar cells [3], highly sensitive sensors [4], and conductive coatings for lightning strike protection and electromagnetic interference shielding [5] due to excellent physical, chemical and mechanical properties [6], such as being light-weight, having a high specific stiffness and specific strength, a high electrical/thermal conductivity, and transparency.

The spatial dispersion state of the fibers in the matrix is a core feature of composites in addition to the intrinsic properties of fibers, such as aspect ratio [7,8] and curliness [9], and plays an important role in tuning the macro properties [10,11]. Generally, increasing the uniformity of the dispersion of fibers would improve the material properties related to the connectivity of the fiber network [7,12,13]. For example, fibers with higher uniformity of dispersion are easier to construct a connecting pathway spanning through the whole materials for conducting electricity, transferring heat, and carrying a load, leading to a lower percolation threshold [14]. However, even when the dispersion state can be improved by using mechanical methods [15] (e.g., sonication, ball milling, and shear mixing) or chemical methods [16] (e.g., surfactants and functionalization methods), re-aggregation of fillers in the subsequent processing (e.g., curing processing) may occur due to the entanglement and interaction between the fillers [7,11,17,18]. Although aggregation could slightly facilitate local electron transfer by enhancing fiber-to-fiber contact [19,20,21], it can reduce the global connectivity of fiber networks and thus increase the percolation threshold, significantly degrading the electrical property of composites [7,12,22,23,24,25]. Therefore, the effect of fiber aggregation on composite properties is crucial and should be carefully treated in the process of material design and manufacturing. 

Before investigating the effect of fiber aggregation on composite properties, it is imperative to quantitatively evaluate the degree of aggregation. The microstructure of nanofibers in composites usually can be characterized by microscopies and image analysis. It is observed that the nanofibers can aggregate into lump-like clusters [19,26,27] or network-like structures with the boundaries of clusters overlapped [28], with different fabrication techniques. When the degree of aggregation is relatively high, the aggregating clusters can be distinguished clearly in the microscopic images, and thus the degree of aggregation usually can be evaluated by the features of aggregating clusters. One of the widely used characterizing methods for the degree of aggregation is based on two indices [17,26], e.g., the variations of location and the size of aggregating clusters. A typical example is the quadrat method based on image processing. With a preliminary grid division of the microscopic image of the composite sample, the standard deviation of the clusters’ area per grid, as well as the characteristic parameter of distribution law for the size of clusters, can be abstracted to describe the degree of aggregation [26]. Most of these methods need a division of the sample image in advance. The most noticeable inconvenience of these quadrat methods is that the precision and reliability of the calculation largely depend on the grid size and number of aggregating clusters [17,26]. Although some work has been performed to remove the effect of the grid size and the number of aggregating clusters to obtain reliable results [17], these methods should be used “with caution” [17,29,30]. Additionally, it is inconvenient to evaluate the overall dispersion quality of samples when using a multi-parameter model, which would lead to a decision with subjectivity [29]. Therefore, some efforts have been made by using measures with a single index [31,32,33], which are based on statistical parameters, such as entropy [31,32] and energy [33]. These indices can quantitatively evaluate the degree of aggregation, but are not easy to obtain due to their complex definitions or mathematics [17]. When the degree of aggregation is relatively small, the nanofibers in the composites present network-like structures, where the boundaries of the aggregating clusters are hard to distinguish due to partial overlap. Therefore, the local features at the level of fibers should be considered to evaluate the degree of aggregation. To this end, some studies have employed multi-level models with the local features of fibers to investigate the electrical property of nanofiber composites, where the aggregating clusters are assumed to have the same size and distribute randomly or regularly in the matrix [7,11,22,23,34,35]. Based on the multi-level model, a typical evaluation for the degree of aggregation is using three indices, i.e., the nominal radius of aggregating clusters, the volume fraction of nanofibers in each aggregating cluster, and the ratio of aggregated nanofibers to the total nanofibers [7,34]. From all the above, it can be found that an evaluation of the degree of aggregation that uses fewer indices and applies to different aggregation topologies remains insufficiently explored. 

Therefore, this paper aims to establish a simple and universal index to quantify the aggregating state of fibers in nanocomposites and further establish the relationship between this index and the percolation threshold. The results of this work may provide a guide to the property characterization, performance prediction, and material design of nanocomposites, and give physical insight into the understanding of systems with similar non-uniform distributions.

## 2. Model and Methods

### 2.1. Aggregation Degree

An index for characterizing the aggregation degree of nanofiber-reinforced composites should be simple, universal, and easy to characterize the material properties. To achieve this purpose, an ideal aggregation degree should have the following three features, simultaneously.

1. The aggregation degree should be a single dimensionless index with physical meaning.
2. The aggregation degree should be applicable to different aggregation topologies, from lump-like aggregating clusters to network-like aggregating clusters.
3. The aggregation degree should have a one-to-one corresponding relation with the electrical property of the composites regardless of the distribution law of fibers.

To obtain an index that can have the above features and facilitate the evaluation of the effect of fiber aggregation on the electrical properties of composites (the percolation threshold is focused on in this work), the key point is to find a parameter closely related to the dispersion state of fibers. Meanwhile, this parameter should be able to reflect the feature at the level of fiber, and thus can apply to different aggregation topologies. It is well-known that the distribution state of the fibers can be reflected by the average number of fibers that have interacted with each fiber [36,37], also known as the average intersection number (softcore fiber) or average bond number (hardcore fiber). This number can be derived from the probability of two arbitrary fibers intersecting (softcore fiber) or making contact (hardcore fiber) with each other. Meanwhile, for composites with uniformly distributed fibers, i.e., the center points of fibers distribute evenly in the space and the orientation angles of fibers follow uniform distributions in the ranges of [0, π), the average interaction number of each fiber is a well-accepted and powerful parameter to theoretically predict the percolation threshold [38,39]. Obviously, when the fibers cannot disperse uniformly in the matrix, the probability for two arbitrary fibers to intersect or contact with each other would change, and the average interaction number on each fiber would also change accordingly. Therefore, it is reasonable to assume that the aggregation degree is related to the variation quantity of the average interaction number, and is defined as:

(1) $$\xi ={\bar{N}}_{\mathrm{int}}-{\bar{N}}_{\mathrm{int}}^{R}$$

where ${\bar{N}}_{\mathrm{int}}$ is the average interaction number of fibers with aggregation and ${\bar{N}}_{\mathrm{int}}^{R}$ is the average interaction number of the same fibers with an assumed uniformly distributed state (i.e., without aggregation). In other words, *ξ* is a shifted average interaction number of fibers. It can be seen from the definition that the aggregation degree has the first feature.

According to the definition in Equation (1), the aggregation degree of fibers is an index that represents a deviation from an assumed state when the same fibers are uniformly distributed. For fibers with uniform distribution, it equals 0. For simplicity, the softcore fiber model [4,36,37,40] is employed here, where the penetration between fibers is permitted. Therefore, the fibers can intersect with each other, and the average interaction number is the average intersection number. The average intersection number of fibers with uniform distribution in a 2-dimensional (2D) space can be obtained by geometric probability analysis [38] and can be expressed as:

(2) $${\bar{N}}_{\mathrm{int}}^{R}=p^{R}\cdot N_{f}=\frac{2}{\pi }\frac{l_{f}^{2}}{L^{2}}N_{f}$$

where *p*^R^ is the probability of two arbitrary fibers intersecting with each other in this case, *l*_f_ is the length of the fiber, *L* is the length of squared representative area element (RAE), and the *N*_f_ is the number of fibers in this RAE. A detailed derivation of Equation (2) can be found in the previous work [38]. For the model with fiber aggregation, due to the non-single-level feature induced by aggregation, it is hard to obtain the average intersection number ${\bar{N}}_{\mathrm{int}}$ by conventional geometric probability analysis and, thus, a two-level analysis to calculate the average intersection number will be introduced in Section 2.2. 

### 2.2. Analysis of the Average Intersection Number 

To analyze the average intersection number of fibers with aggregation, a two-level model based on the softcore fiber network is set up first. The two-level model has been used to predict electrical properties [7,23,34], as well as mechanical properties, such as Young’s modulus [41]. Then, the average intersecting probability between the two fibers is derived based on the model. 

#### 2.2.1. Two-Level Aggregation Model

**Figure 1:**
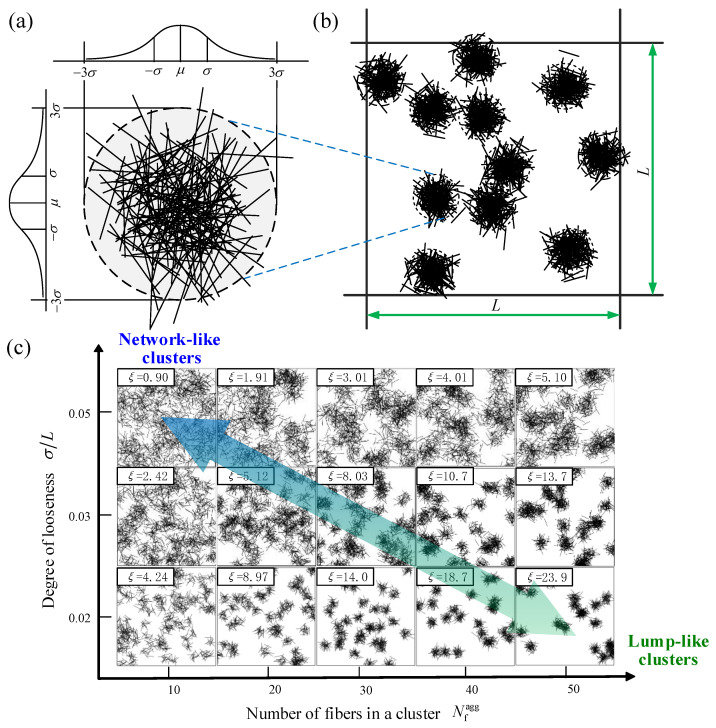
Schematic diagrams of (**a**) an aggregating cluster, (**b**) an RAE with multiple aggregating clusters, and (**c**) RAEs with different fiber dispersion states.

As a widely used methodology, the aggregation model can be established based on the topological feature extracted from microscopy images of nanofiber composites with aggregation [7,16,17]. According to the previous experimental and theoretical studies [26,28,42], the fibers in the composites usually aggregate into many aggregating clusters, and thus a two-level model [7,43] is built as follows. In the micro level of the aggregating cluster, the center points of the fibers follow a normal distribution in two perpendicular directions [23,43] and the orientation angles of fibers follow uniform distributions in the ranges of [0, π), as shown in Figure 1a. Here, *σ* is the standard deviation of a normal distribution which characterizes the degree of looseness. The characteristic radius of the aggregating cluster is considered to be 3*σ*, which includes 99.7% of the fibers. In the macro level of the composites, there are multiple aggregating clusters distributing in the RAE with the size of *L* × *L*, as shown in Figure 1b. 

For simplicity, we assumed that all aggregating clusters in an RAE have the same degree of looseness *σ* and fiber number $N_{f}^{\mathrm{agg}}$ (other distribution laws will be discussed in Section 3.3). Thus, the number of aggregating clusters in an RAE is:

(3) $$N^{\mathrm{agg}}=\frac{N_{f}}{N_{f}^{\mathrm{agg}}}.$$

In addition, when the fiber number in each aggregating cluster $N_{f}^{\mathrm{agg}}$ is 1, the aggregation model can degrade to the uniform distribution model. Additionally, to evaluate the concentration of the fibers, the relative density is defined as the area fraction of the fibers in an RAE [38], as follows:

(4) $$\rho =\frac{N_{f}l_{f}d_{f}}{L^{2}}=\frac{n_{f}l_{f}^{2}}{\lambda_{f}},$$

where *d*_f_ is the diameter of the fiber, *λ*_f_ = *l*_f_/*d*_f_ is the aspect ratio of the fiber, and *n*_f_ = *N*_f_/*L*^2^ is the number of fibers per unit area. For 2D networks, the diameter effect can be ignored because the aspect ratio of nanofibers is usually sufficiently large (e.g., for carbon nanotubes, the aspect ratio is generally larger than 100 [44,45]), and thus the combined dimensionless parameter *n*_f_$l_{f}^{2}$, which is the average fiber number in the area of *l*_f_ × *l*_f_, also can be used to describe the density of fiber network.

As shown in Figure 1c, RAEs with various dispersion states of fibers are exhibited. The aggregation degree *ξ* of each RAE is calculated by Equation (1), as presented in the labels in Figure 1c. It can be seen intuitively that the fibers with stronger aggregation have a larger value of *ξ*. Meanwhile, it also can be found that the aggregation degree has a second feature, i.e., it applies to both lump-like clusters to network-like clusters. 

#### 2.2.2. Intersecting Probability in an Aggregating Cluster

**Figure 2:**
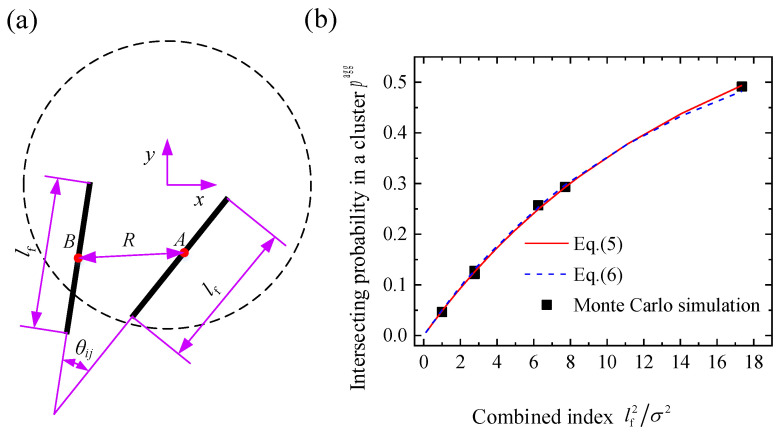
(**a**) A schematic diagram of two arbitrary fibers in an aggregating cluster and (**b**) the intersecting probability vs. combined index ($l_{f}^{2}$/*σ*^2^).

First, to calculate the intersecting probability of two arbitrary fibers in an individual aggregating cluster, a Cartesian coordinate system is introduced whose origin is located at the center of a characteristic circle, as shown in Figure 2a. The intersecting probability of the the two fibers depends on the distance *R* between the midpoints of the two fibers and the angle *θ_ij_* between them. As the distance *R* decreases, or the angle *θ_ij_* approaches π/2, the intersecting probability increases. Based on geometric probability analysis, the intersecting probability of the two fibers in an aggregating cluster is expressed as:

(5) $$p^{\mathrm{agg}}=\iint G(R,\theta_{ij})\cdot f(R)\cdot f(\theta_{ij})dRd\theta_{ij},$$

where *f*(*R*) and *f*(*θ_ij_*) are the probability density functions of the distance *R* and angle *θ_ij_*, respectively, and *G*(*R*, *θ_ij_*) is the intersecting probability of the two fibers with given *R* and *θ_ij_*. The details can be found in Appendix A. 

The intersecting probability of the two fibers in an aggregating cluster *p*^agg^ can be obtained by the Newton–Cotes numerical integration based on Equation (5). By fitting the numerical results of Equation (5), an approximate formulation of the intersecting probability in an aggregating cluster can be expressed as:

(6) $$p^{\mathrm{agg}}\approx \frac{2}{\pi }\left[1-\exp\left(-0.08{\left(l_{f}/\sigma \right)}^{2}\right)\right].$$

To validate the theoretical result of Equation (5) and the approximate solution of Equation (6), Monte Carlo simulations [38] are used to obtain the intersecting probability of the two fibers in an aggregating cluster. As shown in Figure 2b, the numerical results from cases with different degrees of looseness *σ* and the fiber length *l*_f_ fit well with the theoretical result of Equation (5) and the approximate formulation in Equation (6).

#### 2.2.3. Intersecting Probability in an RAE 

In this section, the intersecting probability of the two fibers in an RAE is estimated. Obviously, the intersecting probability of two arbitrary fibers in an RAE *p* is a function of that in an aggregating cluster *p*^agg^. To derive the relation between *p* and *p*^agg^, an RAE with two aggregating clusters is taken as an introductory example first. The intersecting probability of the two fibers in this RAE can be written as:

(7) $$p=\frac{2N_{\mathrm{int}}}{N_{f}^{2}}=\frac{2p^{\mathrm{agg}}{\left(N_{f}^{\mathrm{agg}}\right)}^{2}+2N_{\mathrm{int}}^{\mathrm{add}}}{{\left(2N_{f}^{\mathrm{agg}}\right)}^{2}},$$

where *N*_int_ is the total number of intersections and $N_{\mathrm{int}}^{\mathrm{add}}$ is the number of intersections caused by intersecting fibers from two different aggregating clusters. Because the size of an aggregating cluster is typically much smaller than the RAE, the fibers from two different aggregating clusters can hardly intersect with each other. Therefore, the additional intersection number $N_{\mathrm{int}}^{\mathrm{add}}$ can be ignored compared to a much larger total intersection number *N*_int_, and then the probability in Equation (7) can be simplified as *p* ≈ *p*^agg^/2.

Therefore, it is intuitive that the intersecting probability must satisfy three special conditions, which are: (1) when *N*^agg^→∞, *p*→*p*^R^; (2) when *N*^agg^→1, *p*→*p*^agg^/*N*^agg^; and (3) when *N*^agg^ = 1, *p* = *p*^agg^. Then, the relation between *p* and *p*^agg^ is proposed as:

(8) $$p=\frac{p^{\mathrm{agg}}-p^{R}}{N^{\mathrm{agg}}}+p^{R}.$$

**Figure 3:**
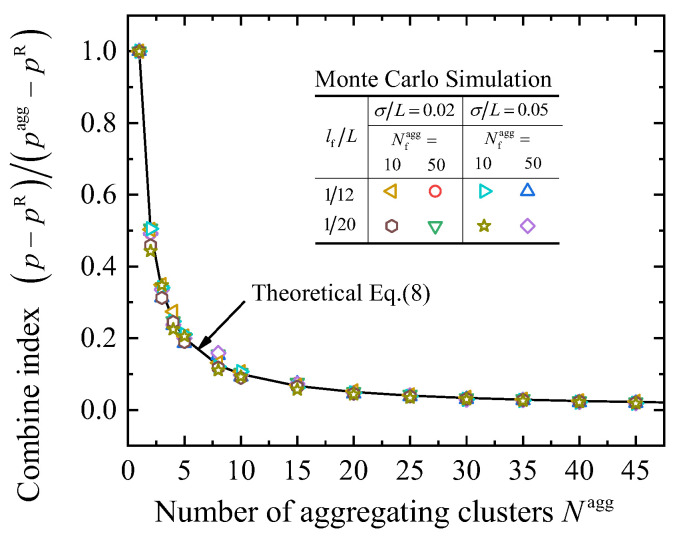
Validation of the average intersecting probability.

To validate the function in Equation (8), Monte Carlo simulations are conducted by using RAEs with various parameters to numerically obtain the intersecting probability *p*. There are three parameters that affect the average intersecting probability, i.e., the degree of looseness *σ* and the fiber length *l*_f_, and the number of fibers in an aggregating cluster $N_{f}^{\mathrm{agg}}$. Therefore, a comprehensive validation is summarized as shown in Figure 3, which shows a good agreement between the Monte Carlo simulation results and Equation (8). 

The average intersection number of each fiber in an RAE finally can be obtained as:

(9) $${\bar{N}}_{\mathrm{int}}=p\cdot N_{f}.$$

### 2.3. Monte Carlo Simulations on the Percolation Threshold 

The percolation threshold can also be obtained by Monte Carlo simulations [8,46,47,48]. In this study, a 2D softcore fiber network model was used to predict the percolation threshold [22], which has been proven to be in good agreement with experimental results [49]. The two-level aggregation model proposed in Section 2.2.1 was used. Percolation occurs when a connecting path that spans through the RAE is formed. Geometric (structural) percolation and electrical percolation are considered to occur simultaneously [36,37,50]. Therefore, in the simulation, RAE samples of fiber networks were generated, and then whether connecting paths are formed in each sample was checked. For a given set of parameters, the simulation can be repeated sufficiently large times (500 times in this work) to obtain a converged value of connection probability. With the increase in network density, an S-shaped sharp change from 0 to 100% can be captured for the connection probability, and the tendency can be well described by the Boltzmann function. It has been proven that the network density when the connection probability is 50% it can be used to estimate the percolation threshold. Additionally, an RAE size of *L*/*l*_f_ ≥ 12 is used to achieve numerical convergence [23]. More details of the Monte Carlo simulation on the percolation threshold can be found in Appendix B.

## 3. Results and Discussion 

### 3.1. Results of the Aggregation Degree 

Based on the analysis of the intersecting probability and the average intersection number in Section 2.2, the aggregation degree of an RAE will be calculated in the following. According to Equations (1)–(4) and (9), the aggregation degree can be expressed as:

(10) $$\xi =\left(p^{\mathrm{agg}}-p^{R}\right)N_{f}^{\mathrm{agg}}.$$

In general, the intersecting probability in aggregating cluster *p*^agg^ is much larger than that in composites with uniform distribution *p*^R^, i.e., *p*^agg^>>*p*^R^, thus Equation (10) can be rewritten as:

(11) $$\xi =p^{\mathrm{agg}}N_{f}^{\mathrm{agg}}=\frac{2}{\pi }\left[1-\exp\left(-0.08{\left(l_{f}/\sigma \right)}^{2}\right)\right]N_{f}^{\mathrm{agg}}.$$

It can be seen in Equation (11) that the aggregation degree is only dependent on the local features of the aggregating cluster (*σ* and $N_{f}^{\mathrm{agg}}$). Additionally, the aggregation degree is almost independent of the density of nanofibers in the composites, as shown in Appendix C.

**Figure 4:**
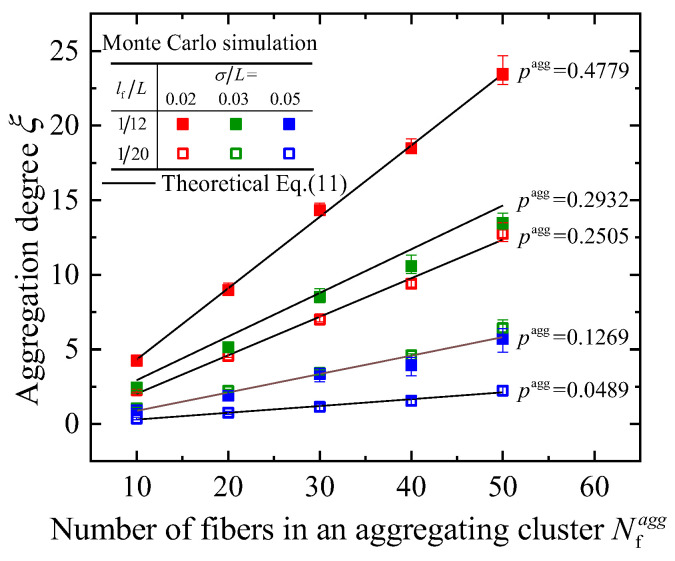
A comparison between the aggregation degree obtained by theory and Monte Carlo simulations (error bars represent the maximum and minimum values of 80 different samples).

A comprehensive comparison between the theoretical results obtained by Equation (11) and numerical results obtained by Equation (1) using Monte Carlo simulations is shown in Figure 4. The theoretical results exhibit good consistency with the results of Monte Carlo simulations. Moreover, it should be noted that different fiber lengths *l*_f_ and degrees of looseness *σ* may also have the same aggregation degree. (e.g., the cases when *p*^agg^ = 0.1259 in Figure 4). The index provides a quantitative description of the degree of fiber aggregation and can be used to qualify the performance of composites by combining quantitative evaluations of specific properties. 

### 3.2. Linear Relation between the Aggregation Degree and the Percolation Threshold

The aggregation degree has been proven to have the first and second features above. Then, does the aggregation degree have the third feature? I.e., is there a one-to-one corresponding relation between the aggregation degree and the percolation threshold regardless of the distribution law of fibers? In this section, the influence of the aggregation degree on the percolation threshold is investigated accordingly. 

Here, the critical area fraction of fibers *ρ*_th_ to trigger the network connectivity, i.e., connection probability is 50%, is used to characterize the percolation threshold. For simplicity, a nominalized threshold ${\hat{\rho }}_{\mathrm{th}}$ is defined as the ratio of the percolation threshold of the aggregation model to that of the uniformly distributed model $\rho_{\mathrm{th}}^{R}$, as:

(12) $${\hat{\rho }}_{\mathrm{th}}=\frac{\rho_{\mathrm{th}}}{\rho_{\mathrm{th}}^{R}},$$

where $\rho_{\mathrm{th}}^{R}$ is proven to be dependent on the aspect ratio of the fibers *λ*_f_. For the 2D model, it can be expressed as [36,38]:

(13) $$\rho_{\mathrm{th}}^{R}=\frac{5.8}{\lambda_{f}}=5.8\frac{d_{f}}{l_{f}}.$$

**Figure 5:**
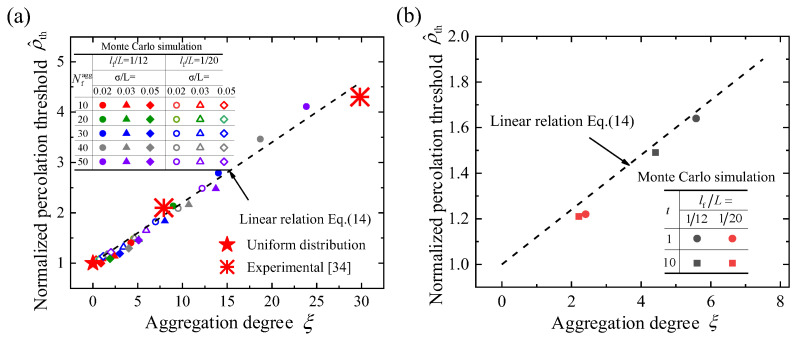
(**a**) The relationship between the aggregation degree and the normalized percolation threshold and (**b**) the relationship between the aggregation degree and the normalized percolation threshold for fibers with different distributions.

Figure 5a shows the relationship between the aggregation degree and the percolation threshold. The normalized percolation threshold increases monotonically with the increase in the aggregation degree, which can be well linearly fitted as:

(14) $${\hat{\rho }}_{\mathrm{th}}=0.12\xi +1.$$

For fiber systems with uniform distribution, i.e., the aggregation degree is 0, the corresponding normalized percolation threshold is 1. For fiber systems with aggregation, the aggregation degree is greater than 0, and the aggregation degree increases with the degree of looseness *σ* and the number of fibers in an aggregating cluster $N_{f}^{\mathrm{agg}}$. It is noted that although models with different parameters may have the same aggregation degree, the aggregation degree has a one-to-one corresponding relation with the threshold. 

### 3.3. Aggregation with Different Distributions

The relationship between the aggregation degree and the percolation threshold discussed above is based on the two-level model, where the randomly distributed aggregating clusters have the same size. However, in reality, the fibers with aggregation may have different distributions, which rises another question: is the linear corresponding relation still applicable to other fiber distributions? To answer this question, a distribution that is extracted from actual cases is built and discussed. In practice, the sizes of the aggregating clusters are diverse, and many studies have found that the frequency of aggregating clusters with a different number of fibers follows a power law [26,28,51]. This indicates that most aggregating clusters include a small number of fibers and aggregating clusters with a larger number of fibers are much fewer. According to the previous studies [26,28], the probability density of $N_{f}^{\mathrm{agg}}$ can be expressed as:

(15) $$f=C{\left(N_{f}^{\mathrm{agg}}\right)}^{-t},$$

where *C* is a constant and *t* is the exponent of the power law. It is assumed that the number of fibers in an aggregating cluster $N_{f}^{\mathrm{agg}}$ is proportional to the degree of looseness *σ*. The rejection sampling method [52] was then used to generate the fiber system with the power-law aggregation distribution. Here, $N_{f}^{\mathrm{agg}}$ and *σ* are in the range of [10, 50] and [0.02, 0.05], respectively. Figure 5b shows the relationship between the aggregation degree and the normalized percolation threshold of the models with the power law slope *t* = −1 and −10. The aggregation degree is calculated by Equation (1). The results show that the linear relation of Equation (14) is still applicable for the fiber system with the power law aggregation distribution. Therefore, it can be concluded that the proposed method in this work is practical and robust, and the aggregation degree has a third feature. 

For composites with aggregation degree *ξ*, the percolation threshold can be obtained from Equations (12)–(14), and expressed as:

(16) $$\rho_{\mathrm{th}}\left(\xi \right)=\left(0.696\xi +5.8\right)\frac{d_{f}}{l_{f}}.$$

By virtue of this relation, the aggregation degree can be used to directly evaluate the properties in regard to percolation. 

### 3.4. Comparison with Experimental Results

The theoretical prediction for the percolation threshold (Equation (14) is compared with the experimental results from the literature [34,53,54]. Two aggregation samples with different parameters are used. Sample 1: the diameter of the aggregating cluster is ~3 μm, the number of fibers in an aggregating cluster is ~30, and the sample has a size of 30 × 30 ${\mu m}^{2}$. Sample 2: the diameter of the aggregating cluster is ~30 μm, the number of fibers in an aggregating cluster is ~1000, and the sample has a size of 300 × 300 ${\mu m}^{2}$. The length of fibers in both samples is ~1.5 μm. The aggregation degrees of the two samples are obtained by Equation (1). The percolation threshold can be obtained by fitting the electrical conductivity of composites at different fiber concentrations with the widely used scaling relation *σ*_ele_ = *σ*_0_(*ρ* − *ρ*_th_)*^n^*, where *σ*_0_ is a constant and *n* is the exponent [55]. As shown in Figure 5a, it is clear that the experimental results agree with the linear relation of theoretical prediction, which validates our model. 

## 4. Conclusions

In this work, a simple and universal single index is proposed to characterize the aggregating state of fibers in nanocomposites, and the effect of fiber aggregation on the percolation threshold is studied based on the proposed index. 

The aggregation degree is defined as a dimensionless single index with a straightforward physical meaning. It is the increment of the average intersection numbers of fibers in composites when fibers aggregate from a uniform distribution state. Based on a two-level model with randomly distributed aggregating clusters, we have demonstrated that the aggregation degree applies to different aggregation topologies, from lump-like aggregating clusters to network-like aggregating clusters, and only depends on the local features of the aggregating clusters by both theoretical geometric probability analysis and Monte Carlo simulations. The index can be concisely expressed as a combination of the intersecting probability and fiber numbers in an aggregating cluster. 

A one-to-one relationship between the aggregation degree and the percolation threshold is found. By using Monte Carlo simulations, the percolation threshold of composites with fiber aggregation is obtained. It is found that the percolation threshold increases monotonously with the increase in the aggregation degree, which can be described by a linear relation. Furthermore, it is proven that this one-to-one linear relation is universally applicable to systems with different distribution laws. 

The new index for the degree of aggregation and its linear relation with the percolation threshold can not only provide a guide to the property characterization, performance prediction, and material design of nanocomposites, but also give a new physical insight into the understanding of a system with complex randomness. 

## Figures and Tables

**Figure A1:**
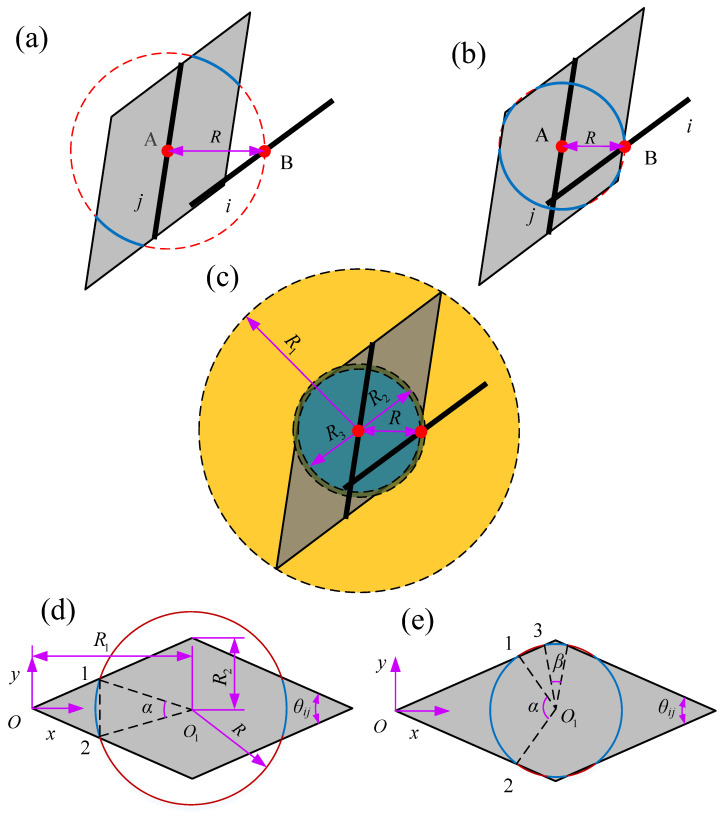
A schematic diagram for the intersecting probability of two arbitrary fibers. (**a**) Four arcs cut by the rhombus edges, (**b**) eight arcs cut by the rhombus edges, (**c**) range partition of four cases of the intersecting probability, and Cartesian coordinate systems of the cases of (**d**) the four arcs and (**e**) the eight arcs.

**Figure A2:**
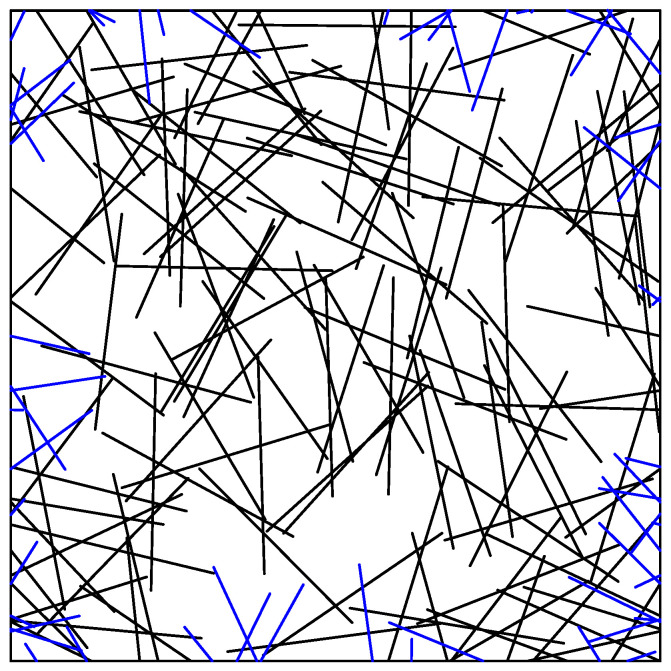
A schematic diagram of the 2D uniformly random nanofiber network after the periodicity process.

**Figure A3:**
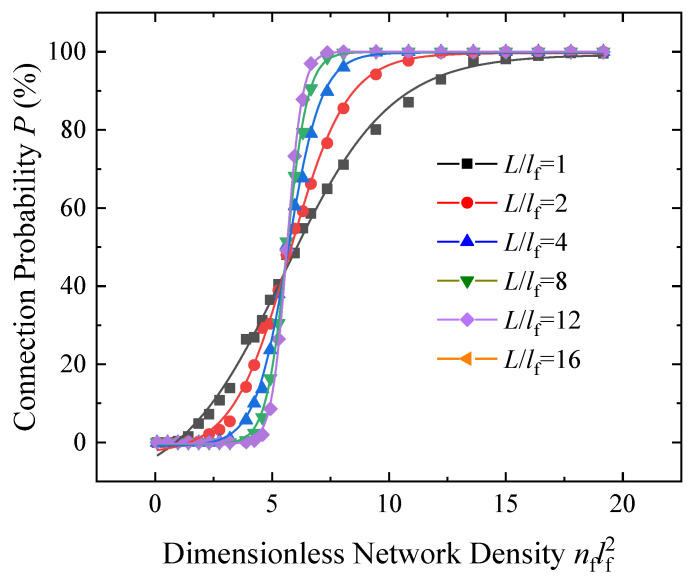
Effect of the relative size of RAE *L*/*l*_f_ on the connection probability *P* of the networks.

**Figure A4:**
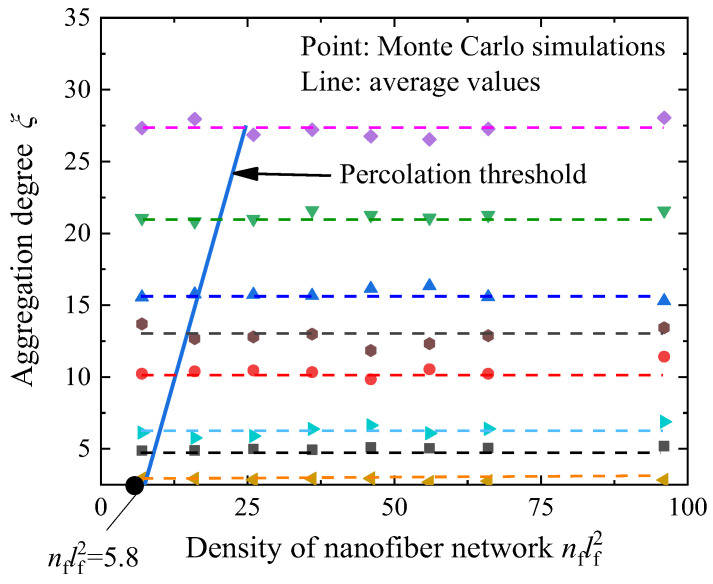
The aggregation degrees of the nanofiber network at different densities.

## Data Availability

All data used to generate these results are available in the main text or Appendix A, Appendix B and Appendix C. Further details could be obtained from the corresponding authors upon reasonable request.

## Appendix A. Intersecting Probability of Fibers

There are three basic unknown functions in Equation (5). *f*(*θ_ij_*) is the probability density function of the angle between two arbitrary fibers. For a fiber system with isotropically distributed angles, it satisfies the following:

(A1) $$f(\theta_{ij})=\frac{2}{\pi }.$$

*f*(*R*) is the probability density function of the center-to-center distance between two arbitrary fibers. To solve *f*(*R*), assuming the coordinates of the midpoints of the two fibers as *A*(*x*_1_, *y*_1_) and *B*(*x*_2_, *y*_2_), as shown in Figure A1a. Then, the distribution function of the center-to-center distance is:

(A2) $$F(R)=\begin{array}{cc} P\left(\sqrt{{\left(x_{2}-x_{1}\right)}^{2}+{\left(y_{2}-y_{1}\right)}^{2}}\leq R\right)=1-\exp\left(-\frac{R^{2}}{4\sigma^{2}}\right) & R>0 \end{array}.$$

By derivation, the probability density function of center-to-center distance can be expressed as:

(A3) $$f(R)=\begin{array}{cc} \exp\left(-\frac{R^{2}}{4\sigma^{2}}\right)\cdot \frac{R}{2\sigma^{2}} & R>0 \end{array}.$$

*G*(*R*, *θ_ij_*) is the intersecting probability of the two fibers with a given angle *θ_ij_* and a center-to-center distance *R*. To obtain this probability, the excluded area method is used [36]. The excluded area is the area around an object where the center of another similarly shaped object cannot enter if penetration is not permitted. Therefore, a necessary and sufficient condition for the two fibers to intersect is that the midpoint of fiber *i* enters the excluded area of fiber *j*, and vice versa. As shown in Figure A1, the gray area is the excluded area of fibers *i* and *j*. When the center of fiber *i* moves along a circle with a radius *R* and enters into the excluded area, these two fibers must intersect each other. Therefore, the intersecting probability *G*(*R*, *θ_ij_*) is the ratio of the arc length inside the excluded area (the blue arcs in Figure A1a,b) to the entire cycle circumference with a radius *R*.

When the angle between the two fibers is fixed, with the radius *R* increasing, the analysis of intersecting probability can be divided into four cases, as shown in Figure A1c.

Case 1: when $0<R\leq R_{3}$, the center of fiber *i* must locate in the excluded area, which means that the two fibers must intersect.

Case 2: when $R_{3}<R\leq R_{2}$, the circle is cut into eight arcs by the rhombus edges, and four arcs are in the rhombus, as shown in Figure A1b.

Case 3: when $R_{2}<R\leq R_{1}$, the circle is cut into four arcs by the rhombus edges, and two arcs are in the rhombus, as shown in Figure A1a.

Case 4: when $R>R_{1}$, the two fibers cannot intersect.

Based on geometric analysis, *R*_1_ is half of the long diagonal of the rhombus, *R*_2_ is the half of the short diagonal of the rhombus, and *R*_3_ is the radius of the inscribed circle of the rhombus. Therefore, *R*_1_, *R*_2_, and *R*_3_ can be written as:

(A4) $$\begin{array}{l} R_{1}=\frac{\sqrt{2}}{2}l_{f}\sqrt{1+\cos\theta_{ij}}\\ R_{2}=\frac{\sqrt{2}}{2}l_{f}\sqrt{1-\cos\theta_{ij}}\\ R_{3}=\frac{\sqrt{2}}{2}l_{f}\sqrt{1+\cos\theta_{ij}}\sin\frac{\theta_{ij}}{2} \end{array}.$$

As mentioned above, the intersecting probability *G*(*R*, *θ_ij_*) is the ratio of the length of blue arcs to the entire cycle circumference. To measure the length of these blue arcs for case 3 and case 4, a Cartesian coordinate system is introduced with the origin located at a vertex of the acute angle of the rhombus, as shown in Figure A1d,e. Obviously, the ratios of the arc lengths can be equivalent to the ratios of angles. Therefore, the piecewise intersecting probability is:

(A5) $$G(R,\theta_{ij})=\left\{ \begin{array}{l} \begin{array}{cc} 1 & \;\;\;\;\;0\leq R\leq R_{3} \end{array}\\ \begin{array}{cc} \frac{\alpha +\beta }{\pi } & R_{3}<R\leq R_{2} \end{array}\\ \begin{array}{cc} \frac{\alpha }{\pi } & \;\;\;\;R_{2}<R<R_{1} \end{array}\\ \begin{array}{cc} 0 & \;\;\;\;\;R\geq R_{1} \end{array} \end{array}\right.,$$

where *α* and *β* are the corresponding angles of blue arcs, and can be expressed as:

(A6) $$\begin{array}{l} \alpha =\arccos\left(1-\frac{2y_{2}^{2}}{R^{2}}\right)\\ \beta =2\arccos\left(\frac{y_{1}}{R}\right) \end{array},$$

where

(A7) $$\begin{array}{l} y_{1}=\frac{R_{2}\left(R_{1}^{2}+\sqrt{R^{2}R_{1}^{2}+R^{2}R_{2}^{2}-R_{1}^{2}R_{2}^{2}}\right)}{R_{1}^{2}+R_{2}^{2}}\\ y_{2}=\frac{R_{2}\left(R_{1}^{2}-\sqrt{R^{2}R_{1}^{2}+R^{2}R_{2}^{2}-R_{1}^{2}R_{2}^{2}}\right)}{R_{1}^{2}+R_{2}^{2}} \end{array}.$$

Finally, according to Equations (5), (A1), (A3) and (A5), the intersecting probability of arbitrary fibers in an aggregating cluster can be rewritten as:

(A8) $$\begin{array}{l} p^{\mathrm{agg}}=\int_{0}^{\pi /2}\int_{0}^{R_{3}}\frac{1}{\pi }\cdot \exp\left(-\frac{R^{2}}{4\sigma^{2}}\right)\cdot \frac{R}{\sigma^{2}}dRd\theta_{ij}\\ \;\;\;+\int_{0}^{\pi /2}\int_{R_{3}}^{R_{2}}\frac{\alpha +\beta }{\pi^{2}}\cdot \exp\left(-\frac{R^{2}}{4\sigma^{2}}\right)\cdot \frac{R}{\sigma^{2}}dRd\theta_{ij}\\ \;\;\;+\int_{0}^{\pi /2}\int_{R_{2}}^{R_{1}}\frac{\alpha }{\pi^{2}}\cdot \exp\left(-\frac{R^{2}}{4\sigma^{2}}\right)\cdot \frac{R}{\sigma^{2}}dRd\theta_{ij} \end{array}.$$

## Appendix B. The Process and Size Effect of the Monte Carlo Simulation on the Percolation Threshold

The process of the Monte Carlo simulation on the percolation threshold is carried out as follows.

Step 1. Generation of the 2D Network Models

In an RAE with a size of *L* × *L*, fibers are simplified as line segments with the length *l*_f_. For a random network without aggregation, the position of the nanofiber midpoint (*x*^0^, *y*^0^) and the orientation of the fiber *θ* follow uniform distributions in the ranges of [0, *L*), [0, *L*), and [0, π), respectively, and follow the equations as:

(A9) $$\{\begin{array}{c} x_{i}^{0}=\mathrm{rand}\times L\\ y_{i}^{0}=\mathrm{rand}\times L\\ \theta_{i}=\mathrm{rand}\times \pi \end{array},$$

where ($x_{i}^{0}$, $y_{i}^{0}$) and *θ_i_* indicate the midpoint position and the orientation angle of the *i*-th fiber in the network, respectively, and the “rand” is a random number uniformly distributed in the range of [0, 1).

For the network with aggregation, the aggregation degree of the networks can be controlled by the degree of looseness *σ* and fiber number $N_{f}^{\mathrm{agg}}$ in an aggregating cluster. The midpoint positions of *N*^agg^ aggregating clusters (*x*^agg^, *y*^agg^) are assumed to be uniformly distributed in the ranges of [0, *L*) and [0, *L*), respectively. The positions of nanofibers in each aggregating cluster are assumed to follow a normal distribution in two perpendicular directions and can be shown as:

(A10) $$\{\begin{array}{c} x_{i}^{0}=x_{j}^{\mathrm{agg}}+\mathrm{normrnd}\left(0,\sigma \right)\\ y_{i}^{0}=y_{j}^{\mathrm{agg}}+\mathrm{normrnd}\left(0,\sigma \right) \end{array},$$

where ($x_{j}^{\mathrm{agg}}$, $y_{j}^{\mathrm{agg}}$) signifies the midpoint position of the *j*-th aggregating cluster. The “normrnd” refers to a random number that conforms to the normal distribution *N*~(0, *σ*^2^). The orientation of the fibers *θ* still follows uniform distribution in the range of [0, π).

The coordinates of two ends of the *i*-th nanofiber can be set as:

(A11) $$\left\{\begin{array}{c} x_{i}^{1}\\ x_{i}^{1} \end{array}\right\}=\left\{\begin{array}{c} x_{i}^{0}\\ x_{i}^{0} \end{array}\right\}+\frac{l_{f}}{2}\left\{\begin{array}{c} \cos\theta_{i}\\ \sin\theta_{i} \end{array}\right\},\left\{\begin{array}{c} x_{i}^{2}\\ x_{i}^{2} \end{array}\right\}=\left\{\begin{array}{c} x_{i}^{0}\\ x_{i}^{0} \end{array}\right\}-\frac{l_{f}}{2}\left\{\begin{array}{c} \cos\theta_{i}\\ \sin\theta_{i} \end{array}\right\}.$$

Assuming the squared RAE is a periodic section in the whole network, the parts of nanofibers outside the area *L* × *L* should be moved to the opposite edge of the RAE boundary, as shown in Figure A2, in which the blue line segments are those moved from outside to inside. The 2D network models of RAE with different aggregation degrees are shown in Figure 1c.

Step 2. Search for the Connecting Path

Step 2.1. Pre-Process

(1) Search the fibers intersected with the left and right edges of the RAE and put them into two groups, i.e., the “input group” and “output group”, respectively, and put all the fibers outside of the “input group” into the “search group”.

(2) Record intersection relations between fibers and store them in a “link matrix”.

Step 2.2. Search for the Conductive Path

(1) Search the fibers in the “search group” that are intersected with fibers in the “input group” according to the “link matrix”.

- If there is no fiber found, the network is not connected and the search process is stopped.
- If there are fibers found, mark these fibers as “fiber temp”.

(2) Check if there are any fibers in “fiber temp” belonging to the “output group”.

- If there are, the network is connected and the search process is stopped.
- If there are not, move the fibers in “fiber temp” from the “search group” to the “input group”, and go to step (1) of Step 2.2.

Step 3. Calculate the Connection Probability

A total of *N*_S_ models for each set of parameters are generated and calculated. The connection probability of the models is the ratio of the number of samples with connecting path *N*_P_ to the total number of samples *N*_S_, and can be written as:

(A12) $$P=\frac{N_{P}}{N_{S}}.$$

Step 4. Calculate the Percolation Threshold Using Boltzmann Function

Simulation results show that the connection probability *P* increases with the increase in the combined dimensionless network density *n*_f_$l_{f}^{2}$, presenting an “S” shape, as shown in Figure A3. This S-shape curve can be described by the Boltzmann function and is written as:

(A13) $$P=P_{2}-\frac{P_{2}-P_{1}}{1+\exp\left(\frac{\left(n_{f}l_{f}^{2}-C_{0}\right)/dx}{P_{2}-P_{1}}\right)},$$

where *P*_1_ and *P*_2_ are the minimum and maximum values of *P*, and should be set as 0% and 100%, respectively, *C*_0_ is the horizontal coordinate of the center of the central symmetrical Boltzmann curve and satisfies the relation $P|{}_{n_{f}l_{f}^{2}=C_{0}}=\left(P_{1}+P_{2}\right)/2$, and d*x* is the slope at the center point. The network density at connection probability *P* = 50% is taken as the percolation threshold [56].

The dimensionless network density *n*_f_$l_{f}^{2}$ of the uniformly distributed random nanofiber network at connection probability *P* = 50% can be predicted by Equation (A13) as 5.8. Thus, the relative density at the percolation threshold is:

(A14) $$\rho_{\mathrm{th}}^{R}=\frac{5.8}{\lambda_{f}}.$$

It is noted that the process of the Monte Carlo simulation on the percolation threshold for networks with random and aggregated nanofibers is the same, except for the modeling process in Step 1.
